# Calcium disodium hexa­thio­diphosphate(IV) octa­hydrate

**DOI:** 10.1107/S1600536810025304

**Published:** 2010-07-03

**Authors:** Claus Ehrhardt, Mimoza Gjikaj

**Affiliations:** a Institute of Inorganic and Analytical Chemistry, Clausthal University of Technology, Paul-Ernst-Strasse 4, 38678 Clausthal-Zellerfeld, Germany

## Abstract

Single crystals of the title compound, CaNa_2_(P_2_S_6_)·8H_2_O, were obtained by adding calcium hydroxide to an aqueous solution of Na_4_(P_2_S_6_)·6H_2_O. The structure is isotypic with that of its strontium analogue and consists of one Ca^2+^ cation, two Na^+^ cations, one-half of a centrosymmetric (P_2_S_6_)^4−^ anion with staggered confirmation and four water mol­ecules in the asymmetric unit. The crystal structure can be described as being built up from layers of cations and anions extending parallel to (101). Within a layer, each CaO_8_ polyhedron is connected *via* edge-sharing to two NaO_4_S_2_ octa­hedra and to one NaO_2_S_4_ octa­edron. The NaO_4_S_2_ octa­hedra are, in turn, linked with two (P_2_S_6_)^4−^ anions through common corners. Various O—H⋯S hydrogen-bonding inter­actions lead to cohesion of adjacent layers. The Ca^2+^ and one Na^+^ cation are situated on a twofold rotation axis and the second Na^+^ cation is situated on an inversion centre.

## Related literature

For background to thio­diphosphates(IV), including their crystal structures, see: Jörgens *et al.* (2003 ▶); Klingen *et al.* (1973 ▶). For the synthesis of Na_4_(P_2_S_6_)·6H_2_O, see: Fincher *et al.* (1998 ▶). For the isotypic structure of SrNa_2_(P_2_S_6_)·8H_2_O, see: Gjikaj & Ehrhardt (2010 ▶).

## Experimental

### 

#### Crystal data

CaNa_2_(P_2_S_6_)·8H_2_O
*M* *_r_* = 484.49
Monoclinic, 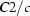

*a* = 14.702 (2) Å
*b* = 9.3081 (14) Å
*c* = 14.052 (2) Å
β = 115.383 (11)°
*V* = 1737.3 (4) Å^3^
*Z* = 4
Mo *K*α radiation
μ = 1.34 mm^−1^
*T* = 223 K
0.29 × 0.24 × 0.23 mm

#### Data collection

Stoe IPDS 2 diffractometer
15223 measured reflections
2650 independent reflections
2369 reflections with *I* > 2σ(*I*)
*R*_int_ = 0.050

#### Refinement

*R*[*F* ^2^ > 2σ(*F* ^2^)] = 0.029
*wR*(*F* ^2^) = 0.065
*S* = 1.13
2650 reflections
122 parameters
All H-atom parameters refined
Δρ_max_ = 0.68 e Å^−3^
Δρ_min_ = −0.53 e Å^−3^

### 

Data collection: *X-AREA* (Stoe & Cie, 2008 ▶); cell refinement: *X-AREA*; data reduction: *X-AREA*; program(s) used to solve structure: *SHELXS97* (Sheldrick, 2008 ▶); program(s) used to refine structure: *SHELXL97* (Sheldrick, 2008 ▶); molecular graphics: *DIAMOND* (Brandenburg, 2004 ▶); software used to prepare material for publication: *SHELXL97*.

## Supplementary Material

Crystal structure: contains datablocks I, global. DOI: 10.1107/S1600536810025304/wm2364sup1.cif

Structure factors: contains datablocks I. DOI: 10.1107/S1600536810025304/wm2364Isup2.hkl

Additional supplementary materials:  crystallographic information; 3D view; checkCIF report

## Figures and Tables

**Table 1:** Selected bond lengths (Å)

Ca—O1	2.4244 (14)
Ca—O2	2.4614 (13)
Ca—O3	2.5123 (13)
Ca—O4	2.5249 (13)
Na1—O3	2.3523 (14)
Na1—O4	2.3713 (13)
Na1—S2^i^	2.9768 (5)
Na2—O2	2.5282 (17)
Na2—S1^i^	2.9242 (8)
Na2—S3	2.9673 (7)
P—S1	2.0156 (6)
P—S2	2.0241 (6)
P—S3	2.0282 (6)
P—P^i^	2.2381 (8)

Symmetry code: (i) 
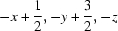
.

**Table 2:** Hydrogen-bond geometry (Å, °)

*D*—H⋯*A*	*D*—H	H⋯*A*	*D*⋯*A*	*D*—H⋯*A*
O1—H1*A*⋯S3^ii^	0.78 (5)	2.58 (5)	3.2922 (17)	153 (4)
O1—H1*B*⋯S2^iii^	0.79 (4)	2.62 (4)	3.2892 (16)	144 (4)
O2—H2*A*⋯S2^ii^	0.79 (3)	2.54 (3)	3.3270 (15)	172 (3)
O2—H2*B*⋯S2^i^	0.88 (3)	2.30 (3)	3.1877 (15)	179 (3)
O3—H3*A*⋯S1^iv^	0.78 (3)	2.43 (3)	3.1908 (15)	168 (2)
O3—H3*B*⋯S1^ii^	0.86 (3)	2.39 (3)	3.2174 (14)	161 (3)
O4—H4*A*⋯S3^v^	0.85 (3)	2.41 (3)	3.2468 (14)	171 (3)
O4—H4*B*⋯S3	0.83 (3)	2.37 (3)	3.1957 (14)	171 (3)

Symmetry codes: (i) 
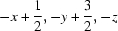
; (ii) 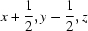; (iii) 
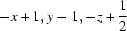
; (iv) 
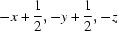
; (v) 
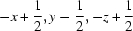
.

**Fig. 1.**
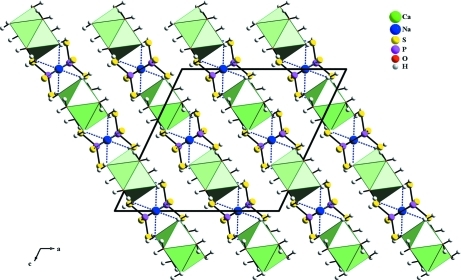
The crystal structure of CaNa2(P2S6).8H2O in a projection along [010].

**Fig. 2.**
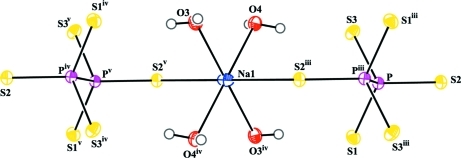
Coordination of Na1 with the atom labelling scheme. The displacement ellipsoids are drawn at the 50% probability level. Symmetry codes as in Table 1. H atoms are represented as spheres of arbirtary radius.

**Fig. 3.**
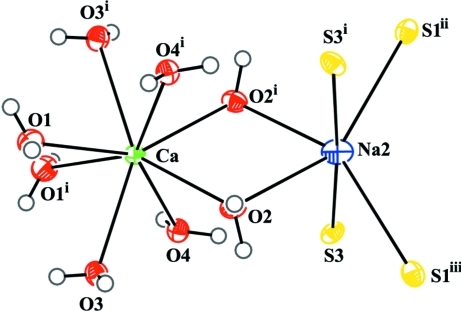
View of the edge-shared CaO8 and Na(2)O2S4 polyhedra with the atom labelling scheme. The displacement ellipsoids are drawn at the 50% probability level. Symmetry codes as in Table 1. H atoms are represented as spheres of arbirtary radius.

## supplementary crystallographic information

### Comment

Alkaline earth hypothiodiphosphates were first reported by Klingen *et al.* (1973). The structure of the title compound is isotypic with that of the strontium analogue, SrNa_2_(P_2_S_6_).8H_2_O (Ehrhardt & Gjikaj, 2010). The asymmetric unit of CaNa_2_(P_2_S_6_).8H_2_O contains one Ca^2+^ cation, two Na^+^ cations, one half of a (P_2_S_6_)^4-^ anion in staggered conformation and four water molecules (Fig. 1).

Na(1) is octahedrally coordinated by four H_2_O molecules and two sulfur atoms of two (P_2_S_6_)^4-^ anions (Fig 2). Na(2) is also octahedrally coordinated by two H_2_O molecules and four sulfur atoms of two (P_2_S_6_)^4-^ anions (Fig. 3). The calcium cation is eightfold coordinated by water O atoms. The [CaO_8_] coordination polyhedron can be described as a bicapped trigonal prism. The crystal structure is built up from layers extending parallel to (101). These layers consists of edge-sharing CaO_8_ and Na(1)O_4_S_2_ polyedra, CaO_8_ and Na(2)O_2_S_4_ polyhadra, as well as corner-sharing Na(1)O_4_S_2_ and (P_2_S_6_)^4-^ polyhedra.

The staggered (P_2_S_6_)^4-^ anion is located on a centre of inversion, with a P—P distance of 2.2381 (8) Å. The P—P central bond links two PS_3_ groups with P—S distances ranging from 2.0156 (6) to 2.0282 (6) Å. These values agree well with those reported previously for another hypothiodiphosphate structure (Jörgens *et al.*, 2003).

Neighbouring layers are held together by various O—H···S hydrogen bonding interactions. The donor—acceptor distances between O atoms of water molecules and S atoms of (P_2_S_6_)^4-^ units range from 3.188 to 3.327 Å (Table 2).

### Experimental

Na_4_(P_2_S_6_).6H_2_O has been prepared according to Fincher *et al.* (1998). The new ternary calcium disodium hexathiodiphosphate octahydrate was obtained by adding calcium hydroxide (2 mmol, 0.148 g) to a solution of Na_4_(P_2_S_6_).6H_2_O (2 mmol, 0.910 g) in 40 ml distilled water at 333 K. Slow cooling to room temperature yielded colorless crystals of the title compound within some days.

### Refinement

Hydrogen atoms were found from the difference Fourier map and were refined independently from their respective oxygen atoms with individual isotropic displacement parameters.

### Crystal data

**Table:**

CaNa_2_(P_2_S_6_)·8H_2_O	*F*(000) = 992
*M**_r_* = 484.49	*D*_x_ = 1.852 Mg m^−^^3^
Monoclinic, *C*2/*c*	Mo *K*α radiation, λ = 0.71073 Å
Hall symbol: -C 2yc	Cell parameters from 15599 reflections
*a* = 14.702 (2) Å	θ = 1.0–30.5°
*b* = 9.3081 (14) Å	µ = 1.34 mm^−^^1^
*c* = 14.052 (2) Å	*T* = 223 K
β = 115.383 (11)°	Block, colorless
*V* = 1737.3 (4) Å^3^	0.29 × 0.24 × 0.23 mm
*Z* = 4	

### Data collection

**Table:**

Stoe IPDS 2 diffractometer	2369 reflections with *I* > 2σ(*I*)
Radiation source: fine-focus sealed tube	*R*_int_ = 0.050
graphite	θ_max_ = 30.5°, θ_min_ = 2.7°
ω–scans	*h* = −20→20
15223 measured reflections	*k* = −13→13
2650 independent reflections	*l* = −20→17

### Refinement

**Table:**

Refinement on *F*^2^	Secondary atom site location: difference Fourier map
Least-squares matrix: full	Hydrogen site location: inferred from neighbouring sites
*R*[*F*^2^ > 2σ(*F*^2^)] = 0.029	All H-atom parameters refined
*wR*(*F*^2^) = 0.065	*w* = 1/[σ^2^(*F*_o_^2^) + (0.0256*P*)^2^ + 2.2311*P*] where *P* = (*F*_o_^2^ + 2*F*_c_^2^)/3
*S* = 1.13	(Δ/σ)_max_ < 0.001
2650 reflections	Δρ_max_ = 0.68 e Å^−^^3^
122 parameters	Δρ_min_ = −0.53 e Å^−^^3^
0 restraints	Extinction correction: *SHELXL97* (Sheldrick, 2008), Fc^*^=kFc[1+0.001xFc^2^λ^3^/sin(2θ)]^-1/4^
Primary atom site location: structure-invariant direct methods	Extinction coefficient: 0.0046 (3)

### Fractional atomic coordinates and isotropic or equivalent isotropic displacement parameters (Å^2^)

**Table:**

	*x*	*y*	*z*	*U*_iso_*/*U*_eq_	
Ca	0.5000	0.24842 (5)	0.2500	0.01531 (10)	
Na1	0.2500	0.2500	0.0000	0.0240 (2)	
Na2	0.5000	0.67523 (13)	0.2500	0.0318 (2)	
P	0.20227 (3)	0.76310 (4)	0.04363 (3)	0.01394 (9)	
S1	0.06893 (3)	0.67248 (4)	−0.04913 (3)	0.02064 (10)	
S2	0.18887 (3)	0.97575 (4)	0.06497 (3)	0.01807 (9)	
S3	0.27954 (3)	0.66563 (4)	0.18473 (3)	0.02051 (10)	
O1	0.59411 (11)	0.04315 (16)	0.23418 (13)	0.0268 (3)	
O2	0.50720 (10)	0.45732 (14)	0.14604 (10)	0.0217 (2)	
O3	0.41702 (10)	0.16862 (14)	0.06107 (10)	0.0216 (2)	
O4	0.31872 (9)	0.32895 (14)	0.17753 (10)	0.0199 (2)	
H1A	0.629 (4)	0.053 (5)	0.205 (4)	0.096 (15)*	
H1B	0.627 (3)	0.002 (4)	0.287 (3)	0.068 (12)*	
H2A	0.554 (3)	0.462 (3)	0.133 (3)	0.048 (9)*	
H2B	0.453 (2)	0.477 (3)	0.088 (3)	0.045 (8)*	
H3A	0.4123 (19)	0.085 (3)	0.059 (2)	0.029 (6)*	
H3B	0.454 (2)	0.192 (3)	0.030 (2)	0.042 (8)*	
H4A	0.288 (2)	0.294 (3)	0.212 (3)	0.049 (8)*	
H4B	0.306 (2)	0.416 (3)	0.172 (2)	0.040 (7)*	

### Atomic displacement parameters (Å^2^)

**Table:**

	*U*^11^	*U*^22^	*U*^33^	*U*^12^	*U*^13^	*U*^23^
Ca	0.0144 (2)	0.01655 (19)	0.01458 (19)	0.000	0.00584 (15)	0.000
Na1	0.0192 (5)	0.0302 (5)	0.0196 (5)	−0.0001 (4)	0.0053 (4)	−0.0011 (4)
Na2	0.0283 (6)	0.0396 (6)	0.0249 (5)	0.000	0.0089 (4)	0.000
P	0.01509 (18)	0.01411 (17)	0.01436 (17)	0.00019 (13)	0.00797 (14)	0.00016 (12)
S1	0.01688 (19)	0.02004 (18)	0.0257 (2)	−0.00401 (14)	0.00981 (15)	−0.00281 (14)
S2	0.01954 (19)	0.01504 (17)	0.01967 (18)	0.00092 (13)	0.00844 (14)	−0.00104 (13)
S3	0.0250 (2)	0.02236 (19)	0.01657 (17)	0.00676 (15)	0.01122 (15)	0.00515 (14)
O1	0.0201 (6)	0.0272 (6)	0.0298 (7)	0.0039 (5)	0.0075 (5)	−0.0023 (5)
O2	0.0197 (6)	0.0261 (6)	0.0187 (5)	−0.0009 (5)	0.0076 (5)	0.0038 (5)
O3	0.0241 (6)	0.0203 (6)	0.0227 (6)	0.0002 (5)	0.0124 (5)	−0.0026 (5)
O4	0.0210 (6)	0.0195 (5)	0.0214 (6)	0.0014 (4)	0.0112 (5)	−0.0002 (4)

### Geometric parameters (Å, °)

**Table:**

Ca—O1	2.4244 (14)	Na2—S1^iv^	2.9242 (8)
Ca—O1^i^	2.4244 (15)	Na2—S3	2.9673 (7)
Ca—O2	2.4614 (13)	Na2—S3^i^	2.9674 (7)
Ca—O2^i^	2.4615 (13)	P—S1	2.0156 (6)
Ca—O3^i^	2.5122 (13)	P—S2	2.0241 (6)
Ca—O3	2.5123 (13)	P—S3	2.0282 (6)
Ca—O4	2.5249 (13)	P—P^iv^	2.2381 (8)
Ca—O4^i^	2.5249 (13)	S1—Na2^iv^	2.9242 (8)
Na1—O3	2.3523 (14)	S2—Na1^vi^	2.9768 (5)
Na1—O3^ii^	2.3523 (14)	O1—H1A	0.78 (5)
Na1—O4	2.3713 (13)	O1—H1B	0.79 (4)
Na1—O4^ii^	2.3713 (13)	O2—H2A	0.79 (3)
Na1—S2^iii^	2.9767 (5)	O2—H2B	0.88 (3)
Na1—S2^iv^	2.9768 (5)	O3—H3A	0.78 (3)
Na2—O2	2.5282 (17)	O3—H3B	0.86 (3)
Na2—O2^i^	2.5283 (17)	O4—H4A	0.85 (3)
Na2—S1^v^	2.9242 (8)	O4—H4B	0.83 (3)
			
O1—Ca—O1^i^	75.99 (8)	O4^ii^—Na1—S2^iii^	90.48 (3)
O1—Ca—O2	113.37 (5)	O3—Na1—S2^iv^	89.02 (4)
O1^i^—Ca—O2	148.04 (5)	O3^ii^—Na1—S2^iv^	90.98 (4)
O1—Ca—O2^i^	148.04 (5)	O4—Na1—S2^iv^	90.48 (3)
O1^i^—Ca—O2^i^	113.37 (5)	O4^ii^—Na1—S2^iv^	89.52 (3)
O2—Ca—O2^i^	75.64 (6)	S2^iii^—Na1—S2^iv^	180.0
O1—Ca—O3^i^	79.99 (5)	O2—Na2—O2^i^	73.30 (7)
O1^i^—Ca—O3^i^	73.01 (5)	O2—Na2—S1^v^	149.72 (4)
O2—Ca—O3^i^	137.37 (4)	O2^i^—Na2—S1^v^	85.08 (3)
O2^i^—Ca—O3^i^	74.41 (4)	O2—Na2—S1^iv^	85.08 (3)
O1—Ca—O3	73.01 (5)	O2^i^—Na2—S1^iv^	149.72 (4)
O1^i^—Ca—O3	79.99 (5)	S1^v^—Na2—S1^iv^	122.01 (5)
O2—Ca—O3	74.42 (4)	O2—Na2—S3	96.20 (4)
O2^i^—Ca—O3	137.37 (4)	O2^i^—Na2—S3	81.01 (4)
O3^i^—Ca—O3	145.61 (6)	S1^v^—Na2—S3	101.16 (2)
O1—Ca—O4	137.92 (5)	S1^iv^—Na2—S3	80.537 (18)
O1^i^—Ca—O4	74.08 (5)	O2—Na2—S3^i^	81.01 (4)
O2—Ca—O4	80.36 (5)	O2^i^—Na2—S3^i^	96.20 (4)
O2^i^—Ca—O4	72.44 (4)	S1^v^—Na2—S3^i^	80.537 (17)
O3^i^—Ca—O4	117.68 (4)	S1^iv^—Na2—S3^i^	101.16 (2)
O3—Ca—O4	73.21 (4)	S3—Na2—S3^i^	176.55 (5)
O1—Ca—O4^i^	74.08 (5)	S1—P—S2	112.06 (3)
O1^i^—Ca—O4^i^	137.92 (5)	S1—P—S3	115.28 (3)
O2—Ca—O4^i^	72.44 (4)	S2—P—S3	109.99 (3)
O2^i^—Ca—O4^i^	80.36 (5)	S1—P—P^iv^	105.34 (3)
O3^i^—Ca—O4^i^	73.21 (4)	S2—P—P^iv^	108.14 (3)
O3—Ca—O4^i^	117.68 (4)	S3—P—P^iv^	105.48 (3)
O4—Ca—O4^i^	145.46 (6)	P—S1—Na2^iv^	105.07 (3)
O3—Na1—O3^ii^	180.0	P—S2—Na1^vi^	137.04 (2)
O3—Na1—O4	78.97 (4)	P—S3—Na2	111.63 (3)
O3^ii^—Na1—O4	101.03 (4)	H1A—O1—H1B	105 (4)
O3—Na1—O4^ii^	101.03 (4)	Ca—O2—Na2	105.53 (5)
O3^ii^—Na1—O4^ii^	78.97 (4)	H2A—O2—H2B	108 (3)
O4—Na1—O4^ii^	179.999 (2)	Na1—O3—Ca	104.38 (5)
O3—Na1—S2^iii^	90.98 (4)	Na1—O4—Ca	103.44 (5)
O3^ii^—Na1—S2^iii^	89.02 (4)	H4A—O4—H4B	106 (3)
O4—Na1—S2^iii^	89.52 (3)		

Symmetry codes: (i) −*x*+1, *y*, −*z*+1/2; (ii) −*x*+1/2, −*y*+1/2, −*z*; (iii) *x*, *y*−1, *z*; (iv) −*x*+1/2, −*y*+3/2, −*z*; (v) *x*+1/2, −*y*+3/2, *z*+1/2; (vi) *x*, *y*+1, *z*.

### Hydrogen-bond geometry (Å, °)

**Table:**

*D*—H···*A*	*D*—H	H···*A*	*D*···*A*	*D*—H···*A*
O1—H1A···S3^vii^	0.78 (5)	2.58 (5)	3.2922 (17)	153 (4)
O1—H1B···S2^viii^	0.79 (4)	2.62 (4)	3.2892 (16)	144 (4)
O2—H2A···S2^vii^	0.79 (3)	2.54 (3)	3.3270 (15)	172 (3)
O2—H2B···S2^iv^	0.88 (3)	2.30 (3)	3.1877 (15)	179 (3)
O3—H3A···S1^ii^	0.78 (3)	2.43 (3)	3.1908 (15)	168 (2)
O3—H3B···S1^vii^	0.86 (3)	2.39 (3)	3.2174 (14)	161 (3)
O4—H4A···S3^ix^	0.85 (3)	2.41 (3)	3.2468 (14)	171 (3)
O4—H4B···S3	0.83 (3)	2.37 (3)	3.1957 (14)	171 (3)

Symmetry codes: (vii) *x*+1/2, *y*−1/2, *z*; (viii) −*x*+1, *y*−1, −*z*+1/2; (iv) −*x*+1/2, −*y*+3/2, −*z*; (ii) −*x*+1/2, −*y*+1/2, −*z*; (ix) −*x*+1/2, *y*−1/2, −*z*+1/2.
